# Chromatic-aberration-corrected diffractive lenses for ultra-broadband focusing

**DOI:** 10.1038/srep21545

**Published:** 2016-02-12

**Authors:** Peng Wang, Nabil Mohammad, Rajesh Menon

**Affiliations:** 1 Department of Electrical and Computer Engineering, University of Utah, Salt Lake City, UT 84112, USA.

## Abstract

We exploit the inherent dispersion in diffractive optics to demonstrate planar chromatic-aberration-corrected lenses. Specifically, we designed, fabricated and characterized cylindrical diffractive lenses that efficiently focus the entire visible band (450 nm to 700 nm) onto a single line. These devices are essentially pixelated, multi-level microstructures. Experiments confirm an average optical efficiency of 25% for a three-wavelength apochromatic lens whose chromatic focus shift is only 1.3 μm and 25 μm in the lateral and axial directions, respectively. Super-achromatic performance over the continuous visible band is also demonstrated with averaged lateral and axial focus shifts of only 1.65 μm and 73.6 μm, respectively. These lenses are easy to fabricate using single-step grayscale lithography and can be inexpensively replicated. Furthermore, these devices are thin (<3 μm), error tolerant, has low aspect ratio (<1:1) and offer polarization-insensitive focusing, all significant advantages compared to alternatives that rely on metasurfaces. Our design methodology offers high design flexibility in numerical aperture and focal length, and is readily extended to 2D.

Recent work has suggested the use of metalenses for broadband achromatic focusing^1^. Here, we show that it is not necessary to invoke concepts of metasurfaces or metalenses to enable such focusing. Scalar diffractive optics, when designed appropriately, can readily enable ultra-broadband achromatic focusing. Such diffractive optics can be far simpler to manufacture and can allow for polarization-independent focusing. An ideal lens focuses one point in the object space to one point in the image space^2^. Almost all imaging systems suffer from chromatic aberrations, which means that light of different wavelengths generate focal spots at different spatial locations^2^. This phenomenon deteriorates the performance of both imaging^3^,^4^ and non-imaging^5^ systems under broadband illumination. For instance, a color camera without chromatic-aberration correction will form spatially displaced and defocused images of the blue, green and red channels.

Chromatic aberration is due to either the dispersion properties of the material or the structure of the optic. For refractive lenses, longer wavelengths focus at a farther distance, since in most dielectric materials, the refractive index decreases at longer wavelengths. Figure 1(a) illustrates the simple example of a bi-convex glass lens and the corresponding shift of its focus, calculated by the Lensmaker’s equation^2^. The conventional diffractive lens (zone-plate), on the other hand, exhibits opposite chromatic aberration (Fig. 1(b))^6^,^7^,^8^. Diffraction angle is proportional to wavelength^2^, and thus longer wavelengths are focused closer than shorter ones.

Chromatic aberration can be corrected approximately by using materials that exhibit complementary dispersion, as in an achromatic doublet and triplet^9^,^10^,^11^. However, this technique is cumbersome, since the number of materials equals the number of wavelengths where the chromatic aberrations are minimized^10^,^11^. The extra alignment makes these lenses expensive and bulky. Hybrid refractive-diffractive lenses perform slightly better, but their complexity is even higher^12^,^13^,^14^. Such designs that work for more than three wavelengths are seldom studied. An alternative approach is to use a phase-coded aperture^15^, but this requires precise polishing of the glass surface. In all these cases, it is challenging to make such corrected lenses with micro-scale thickness.

Metasurfaces exploit surface plasmonic or nanophotonic phenomena to locally impart abrupt phase shift so as to purposely manipulate the diffraction pattern^16^,^17^. Previous studies showed its potential in anomalous reflection and refraction, and complex beam generation^16^,^18^,^19^. Here, we emphasize that metasurfaces are excellent, when the vector properties of light must be manipulated as in the case of a high-efficiency polarizer^20^, but they are not required to manipulate the scalar properties of light. Diffractive optics is a better alternative. The fabrication requirements for metasurfaces are far more stringent in terms of both resolution and precision compared to diffractive optics. Furthermore, metasurfaces are by nature polarization sensitive^1^,^16^,^17^,^18^,^19^,^20^. Here, we reiterate that diffractive optics can readily enable broadband focusing, while still maintaining the planar architecture. Previously, we have functionalized diffractive optics as a solar spectrum splitter/concentrator^21^, multi-color encoder^22^, phase masks for 3D lithography^23^ and dispersion elements in computational spectroscopy^24^.

Here, we extend the concept of broadband diffractive optics to super-achromatic focusing. Specifically, we designed, fabricated and characterized 4 different planar cylindrical chromatic-aberration-corrected lenses. Each lens has a maximum thickness of 3 μm and a minimum feature size of 3 μm. All the devices can be readily patterned using grayscale lithography and inexpensively replicated for mass-production using imprint lithography^25^,^26^. The aberration-correction capabilities of our lenses are on par with or better than commercial doublets. Two types of lenses were constructed. One was designed for three discrete wavelengths, and the other for continuous broadband illumination.

The cross-sectional schematic of our chromatic-aberration-corrected diffractive lens (CACDL) is illustrated in Fig. 2(a). The CACDL is composed of pixels that can be square (2D) or linear grooves (1D). In the devices described here, the grooves are of width, Δ = 3 μm and height, *h*_*i*_ is assigned to the *i*^th^ groove. Each groove imparts a relative phase shift given by 
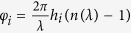
, where *n(λ)* is the refractive index of device material at wavelength *λ*^22^. For simplicity, we utilize a positive-tone photoresist, SC1827 deposited on a soda-lime glass substrate as the device material. A commercial grayscale lithography tool was employed to rapidly pattern the device in a single step^21^,^22^,^23^,^24^. The width, Δ is dictated by the resolution of this tool.

In order to achieve super-achromatic performance, we applied a modified direct-binary-search (DBS) algorithm to optimize the distribution of groove heights, *h*_*i*_^21^,^22^,^23^,^24^,^27^. It is a perturbation-based iterative method. Details of the design algorithm are included in the Supplementary Materials. The target point-spread function (PSF) is defined as a diffraction-limited Gaussian with full-width-at-half-maximum (FWHM) determined by 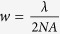. The numerical aperture (*NA*) is given by 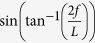 in which *L* = *N*Δ is the total length of the lens, *N* is the total number of grooves, and *f* is the design focal length. Compared to other optimization algorithms for multi-wavelength diffractive optics^28^,^29^, our technique is applicable generally^20^,^21^,^22^,^23^,^24^ and our approach is the first experimental demonstration of super-achromatic and continuous broadband focusing using diffractive optics.

The diffraction pattern in the focal plane is determined by the phase acquired by light transmitting the diffractive lens (Fig. 2(c)) and that acquired via the optical path length in air (Fig. 2(b)). For chromatic-aberration-corrected focusing at *x*′, three or more wavelengths must diffract from location, *x* such that they interfere constructively at the focus, *x*′. Our method for designing the diffractive lens realizes an optimal height distribution that can approximate such constructive interference. In our lens, there are *N* grooves, and each groove can occupy *P* discrete height levels. Therefore, the total degrees-of-freedom can be enormous, *P*^*N*^. This allows extreme design flexibility as illustrated later. We designed and fabricated four CACDLs (see Fig. 2(d)). For each device, we assumed periodic boundaries during design and fabricated 7 periods, each of length *L* = 8.4 mm. The optical micrographs, profilometer measurements of the topography and the scanning-electron micrographs of exemplary devices are shown in Fig. 2(e,f,g), respectively. The cross-sectional micrographs of a cleaved sample (Fig. 2(g)) indicate that the grooves are rounded due to the resolution limitations of our lithography tool. Nevertheless, the average height within each groove was within 100 nm of the design value. It is noteworthy that the maximum aspect ratio is 1:1, which is much smaller than that of the metalenses^1^,^30^. Furthermore, the lithographic resolution required for our CACDLs is only 3 μm (5λ for λ = 600 nm) compared to ~100 nm (0.065λ for λ = 1550 nm) for the metalenses^1^. To achieve broadband focusing in the visible region with the metalenses, one would require features of size 39 nm and aspect ratios of over 3:1.

To experimentally demonstrate chromatic-aberration-corrected focusing, we illuminated each CACDL using a spatially collimated beam from a super-continuum source (SuperK EXTREME EXW6, NKT Photonics) that was first conditioned using a reconfigurable band-pass filter (SuperK VARIA, NKT Photonics). The filter allowed us to illuminate the CACDL with one discrete wavelength at a time (minimum bandwidth 10 nm). Then, a single-mode fiber (SMF, core diameter ~8 μm) connected to a spectrometer (Ocean Optics Jaz) was placed in the vicinity of the designed focus. The fiber was scanned using a motorized 2-axis stage with 3 μm and 10 μm steps along the *X*′ and *Z* axes, respectively. The transmitted spectra were collected at each location. The final results were derived after subtracting the dark spectrum from the raw data and dividing by the reference spectrum (that transmitted through the unpatterned photoresist).

First, we consider the CACDLs designed for three discrete wavelengths (460 nm nm, 540 nm and 620 nm). To demonstrate the flexibility of our approach, we designed 3 different lenses with the following parameters: number of grooves, *N* = 2800, 2800, 280; focal length, *f* = 120 mm, 25 mm and 10 mm, which correspond to numerical aperture, *NA* = 0.035, 0.166 and 0.042, respectively. Figure 3(a–i) summarize the simulated and measured light-intensity distributions in the focal plane at the 3 design wavelengths. As expected all 3 lenses exhibit clear apochromatic focusing. Scalar-diffraction simulations predict average optical efficiencies of 30.0%, 30.4% and 39.0% for the 3 designs. The corresponding measured average optical efficiencies are 24.9%, 23.0% and 21.5%, much higher than those of previously reported achromatic lenses^13^. Even higher efficiency (>50%) is possible with thicker microstructures (Fig. S13 in the Supplementary Materials). In theory, non-ideal efficiency (<100%) is primarily due to lack of perfect interference (constructive at focus and destructive in the background). Generally speaking, this efficiency dictates the contrast or resolution in an optical system. Here, we define the optical efficiency as the ratio of power within the region defined by the first zero to the total incident power. We can also quantify the achromaticity of the CACDLs by measuring the lateral and axial focus shifts as a function of wavelength. These can be calculated by comparing the 2D PSF (*X*′*Z* plane) at each wavelength to that at the center wavelength, 540 nm. The lateral and axial focus shifts for the first design were 0.32 μm, 6.7 μm (simulation) and 1.3 μm, 25 μm (experiment), respectively. These are better than what can be achieved using conventional refractive lens combinations^9^.

Due to the finite diameter of the SMF core, the measured PSFs are wider than the actual distributions. This is especially obvious in the CACDL with the highest NA (Fig. 3(d–f)). Fabrication errors as well as the limited acceptance angle of the SMF contribute to the reduction of optical efficiencies. The 2D PSFs (*X*′*Z*) of the first design at five wavelengths (460 nm, 500 nm, 540 nm, 580 nm and 620 nm) are plotted in Fig. 3(j–n) (simulation) and Fig. 3(o–s) (measurement). The scalar-diffraction simulation has resolution of 0.2 μm and 2.5 μm in *X*′ and *Z* directions. The measured plots are numerically interpolated into the same grid for visual comparison. At the vicinity of the nominal focal plane (white-dashed lines), focusing is clearly observed for only the design wavelengths (460 nm, 540 nm and 620 nm). No focusing is found at the other wavelengths (Fig. 3(k,p,m,r)). Another simple evidence of apochromatic focusing is seen via the images captured at the focal plane using a monochrome sensor (DMM22BUC03-ML, The Imaging Source) with illumination wavelength selected by the VARIA filter, shown as insets in Fig. 3(o–s). Note that the SMF-spectrometer scheme was used to accurately measure PSFs (Fig. 3(a–i) and (o–s)), since the spectrometer has higher spectral resolution (0.4 nm) than the VARIA filter and larger dynamic range (16-bit) than the sensor (8-bit).

Next, we extended our CACDL to focus continuous broadband illumination across the visible spectrum (450 nm–690 nm, super-achromatic). This is achieved by increasing the wavelength sampling to 5 nm during design. It was designed with *N* = 2500, focal length, *f* = 280 mm, and *NA* = 0.013. The simulated and measured 1D PSFs in the design focal plane as a function of wavelength are plotted in Fig. 4(a,b), respectively. Note that the plots are normalized to the peak at each wavelength to account for the spectrum of the source. The white dots (left) and crosses (right) indicate the lateral (Δ*x*) and axial (Δ*f* ) focal-spot shifts in each figure. These shifts were obtained from the 2D (*X*′*Z*) PSFs. The simulated and measured 2D PSFs at 3 wavelengths are illustrated in Fig. 4(d–f) and 4(g–i), respectively. Again, the measurements were interpolated into the same resolution as the simulations. The lateral shift averaged over all wavelengths, 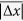 is 0.47 μm (simulation) and 1.65 μm (experiment). The axial shift averaged over all wavelengths, 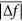 is 23.5 μm (simulation) and 73.6 μm (experiment). Both shifts are significantly smaller than that of a diffractive lens optimized for single wavelength (Fig. S12 in the Supplementary Materials). The maximum axial-focus shift, Δ*f* is comparable to that of commercial achromatic doublets^9^. However, our CACDL is thin (planar), inexpensive and comprised of only a single material. The optical-efficiency spectrum is plotted in Fig. 4(c). The discrepancies between the simulated and measured curves are primarily due to fabrication errors in the CACDL height profile. The efficiency drops at longer wavelengths. This can be potentially compensated by appropriately weighting the efficiencies of different wavelengths during design^21^ and by optimizing the patterning process. As before, monochrome images illuminated by the 3 wavelengths (selected by the VARIA) are shown as insets in Fig. 4(g–i).

The CACDLs are insensitive to the polarization state of the incident light. This is a strong advantage over metalenses, since most imaging systems require polarization-independent focusing. To prove this, we illuminated the first CACDL design (from Fig. 2(a)) with linearly polarized light and observed the focus while the polarization was rotated by 90 degrees. In our nomenclature, the transverse magnetic (TM) refers to electric field polarized along the degenerate direction *Y* of the CACDL, while the transverse electric (TE) refers to that polarized along the *X* direction (see inset of Fig. 5(a)). The measured PSFs for the 3 design wavelengths (Fig. 5(a–c)) are identical for the orthogonal polarizations. Furthermore, finite-difference-time-domain (FDTD) simulations of diffraction by a single groove (Fig. 5(d)) confirm that both amplitude and phase of the diffracted light are identical for both polarizations. This is expected since the smallest period of the CACDL is 6 μm, much larger than the wavelengths of interest.

In all micro-optics, fabrication errors pose an important impact on the optical efficiencies. We numerically analyzed this impact by adding random errors with various standard deviations to the design-height distribution. The results plotted in Fig. 5(e) indicate that the CACDLs are robust to height errors of up to ~100 nm, which, in turn, corresponds to two height levels (Δ*h* = *H*/(*P*−1) = 50 nm). Therefore, our device is relatively tolerant to fabrication errors, which is consistent with previous devices designed using related techniques^20^,^21^,^22^,^23^,^24^. As expected, the efficiency decreases with increasing errors (left *Y* axis in Fig. 5(e)) and the device with fewer grooves (CACDL#3) is more susceptible to fabrication errors^21^,^22^. This is because constructive interference gradually breaks down when the phase distribution deviates from the optimal design. Moreover, the wavelength-averaged axial-focus shift, 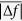 increases with errors (right *Y* axis in Fig. 5(e)). For CACDL#1, 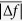 is maintained small when the error is less than 100 nm, while that of the CACDL#4, it deteriorates rapidly. This is likely a consequence of the fact that broadband super-achromatic focusing requires a more stringent phase matching compared to the case of focusing only 3 wavelengths.

We also simulated the impact of oblique incidence (Fig. 5(f)). The wavelength-averaged focus shifts both laterally and axially with change in incident angle, *θ*. Hence, the wavelength-averaged optical efficiency drops with off-normal incidence (top panel). Nevertheless, both studied CACDLs maintain their efficiencies over *θ~*±4^o^. The wavelength-averaged lateral-focus shift, 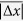 (middle panel) and axial-focus shift, 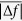 (bottom panel) increase nonlinearly with *θ*. However, both designs preserve reasonable chromatic aberrations over *θ* ~ ±4^o^. Note that even though we assumed periodic boundaries during design, experiments suggest that this is not strictly necessary as elaborated in the Supplementary Materials. Finally, although our devices were 1D, they can be readily extended to 2D^20^,^22^,^23^ and also to almost any electromagnetic spectrum.

## Additional Information

**How to cite this article**: Wang, P. *et al*. Chromatic-aberration-corrected diffractive lenses for ultra-broadband focusing. *Sci. Rep.* **6**, 21545; doi: 10.1038/srep21545 (2016).

## Supplementary Material

Supplementary Information

## Figures and Tables

**Figure 1:**
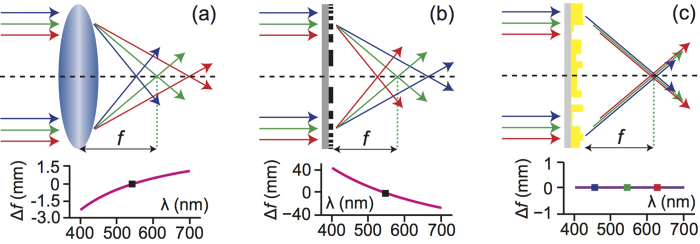
Focusing optics with nominal focal length f = 120 mm at λ = 540 nm (top row) and their calculated axial chromatic aberration Δf (bottom row). Normally incident uniform illumination is assumed. (**a**) Bi-convex refractive lens (BK7 glass). (**b**) Amplitude (binary) zone-plate. (**c**) Schematic explanation of the super-achromatic diffractive lens. Ideally, focus shift over the entire spectrum remains zero.

**Figure 2:**
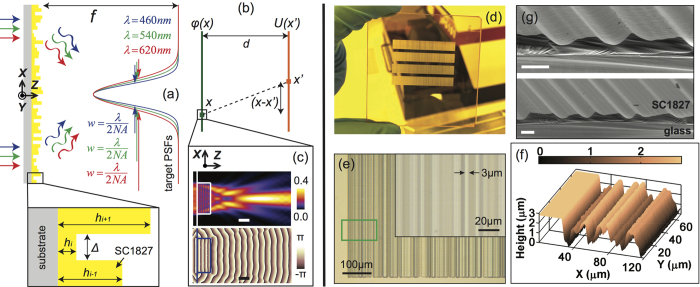
(**a**) Schematic of the chromatic-aberration-corrected diffractive lens (CACDL) with focal length, f. Our first set of CACDLs were designed to focus λ = 460 nm, 540 nm and 620 nm. The desired light-intensity distributions in the focal plane (or the point-spread functions or PSFs) are dictated by diffraction. This 1D CACDL is comprised of linear grooves with a designed height, h_i_. SC1827 is the photoresist used for fabricating the CACDL. (**b**) Illustration of transformation from a CACDL point, x to the focus, x′. (**c**) Intensity (top) and phase (bottom) distributions of light (λ = 540 nm) diffracted by a single groove (width = 3 μm, height = 1.2 μm, scale bars: 1 μm) simulated using FDTD. Linear polarization along X was assumed. (**d**) Photograph of four CACDLs patterned on a glass substrate. (**e**) Optical micrograph of a corner of one CACDL. Inset: magnified view. (**f**) Profilometer image of the region in the green box in (**e**). The maximum height is ~3 μm. (**g**) Scanning-electron-microscopy images of the cross-sections of two CACDLs (scale bars: 5 μm).

**Figure 3:**
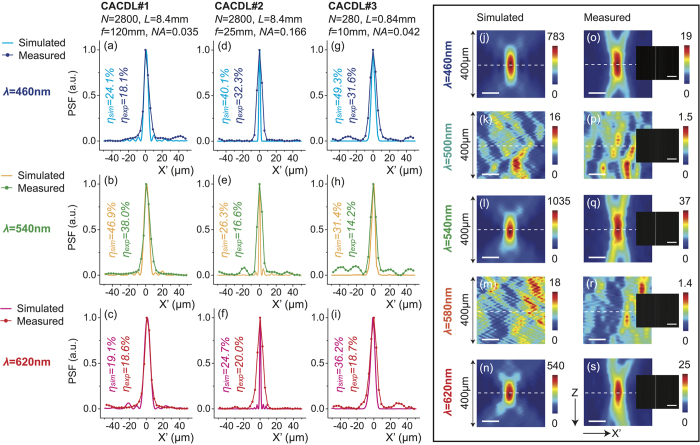
CACDLs for 3 discrete wavelengths (apochromats). The simulated and measured point-spread functions (PSFs) at λ = 460 nm (**a**,**d**,**g**), λ = 540 nm (**b**,**e**,**h**) and λ = 620 nm (**c**,**f**,**i**). Each column represents one CACDL. Simulated (**j**–**n**)) and measured (**o**–**s**) 2D PSFs of the first design for λ = 460 nm (**j**,**o**), 500 nm (**k**,**p**), 540 nm (**l**,**q**), 580 nm (**m**,**r**) and 620 nm (**n**,**s**) (scale bars: 20 μm). Dashed-white lines denote the focal plane. Insets: grayscale images of the focal plane captured by a monochrome CMOS camera when illuminated by the discrete wavelengths from the VARIA filter (scale bars: 1 mm, exposure time = 3 ms).

**Figure 4:**
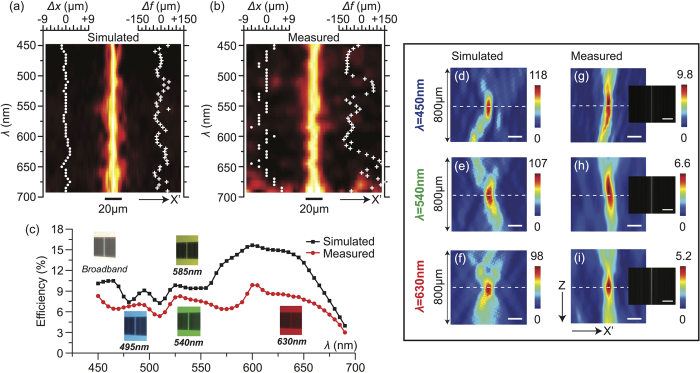
CACDL for broadband (450 nm to 690 nm) focusing (super-achromat). (**a**) Simulated and (**b**) measured 1D PSFs as a function of wavelength. Left insets: lateral-focus shift, Δ*x* versus wavelength (white dots). Right insets: axial-focus shift, Δ*f* versus wavelength (white crosses). (**c**) Simulated (black) and measured (red) optical efficiency as a function of wavelength. Insets: photographs of the focus on a white observation screen at various wavelengths. Simulated (**d–f**) and measured (**g–i**) 2D PSFs for λ = 450 nm (**d**,**g**), λ = 540 nm (**e,h**) and λ = 630 nm (**f,i**) (scale bars: 30 μm). Dashed-white lines delineate the focus. Insets: images of the focus captured by a monochrome sensor (scale bars: 1 mm). Exposure time t = 4 ms.

**Figure 5:**
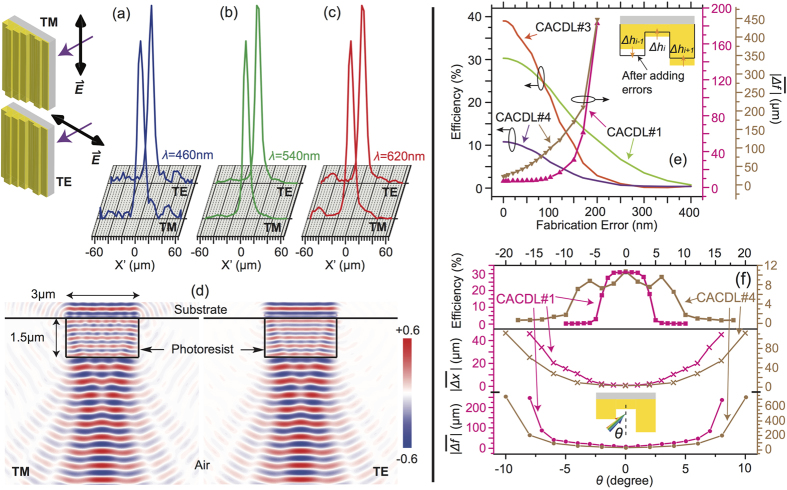
Impact of polarization, fabrication errors and oblique incidence. Measured PSFs of the CACDL#1 at the orthogonal polarizations for (**a**) λ = 460 nm, (**b**) λ = 540 nm and (**c**) λ = 620 nm. Inset: definitions of the incident polarization states. (**d**) Simulated electric-field distribution of light diffracted from one 3 μm-wide and 1.5 μm-high photoresist groove for TM (left) and TE (right) polarizations using FDTD. (**e**) Simulated wavelength-averaged optical efficiency (left Y-axis) and wavelength-averaged axial-focus shift (right Y-axis) as a function of fabrication errors. Inset: schematic showing how fabrication errors are applied. (**f**) Simulated wavelength-averaged optical efficiency (top) and wavelength-averaged lateral-focus shift (middle) and axial-focus shift (bottom) of two CACDLs as a function of the angle of incidence, *θ*. Middle and bottom panels share the same X coordinates. Inset: definition of *θ*.
