# Supplementary material for: Chromatic-aberration-corrected diffractive lenses for ultra-broadband focusing
Source: Sci Rep. 2016 Feb 12;6:21545. doi: 10.1038/srep21545 (PMC4751468; doi:10.1038/srep21545)
Supplement: Supplementary Information [file srep21545-s1.pdf]

Supplementary Materials

Chromatic-aberration-corrected diffractive lenses for ultra-broadband focusing

P. Wang, N. Mohammad and R. Menon

Department of Electrical and Computer Engineering, University of Utah, Salt Lake City, UT 84112, U.S.A.

1. Device simulation model

The generalized geometry of the proposed chromatic-aberration-corrected diffractive lens (CACDL) design is schematically shown in Fig. S1. In the X direction, the diffractive optic (blue grid) is discretized into uniform grooves with width Δx. There is no variation along the Y direction, making it a cylindrical lens. The image space (red grid) is defined in the same grid size.

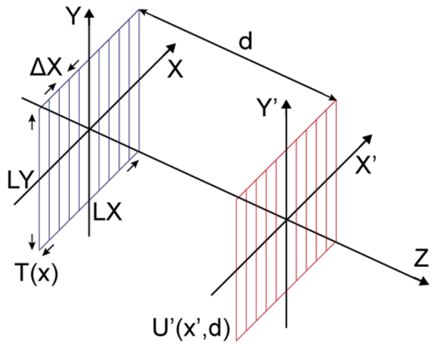

Figure S1 | Schematic illustration of the generalized simulation model of the proposed chromatic-aberration-corrected lens design.

Our devices have groove width Δx =3μm, limited by the resolution constraint of the available lithography tool. The 1D CACDL topography can be described by [1,2]:

$$h(x) = \sum_m \Delta h \cdot p_{(m)} \cdot rect\left(\frac{x - m\Delta x}{\Delta x}\right) \tag{S1}$$

in which $\Delta h = H/(N_{levels}+1)$ is the unit height, H is the maximum height and N_{levels} is the total number of quantization levels; $p_{(m)}$ is the a positive integer within the interval [0, N_{levels}]; *rect( )* is the rectangle function with integer indices m.

Since the CACDL is relatively thin and the imaginary part of the refractive index of the material (SC1827 positive-tone photoresist) within the spectrum of interest (450nm~690nm) is negligibly small, we can reasonably ignore its absorption. Therefore, the transmission function is given by [1,2]:

$$T(x,\lambda) = e^{i\varphi(x)} = 1 + \sum_m rect\left(\frac{x - m\Delta}{\Delta}\right) \cdot \left(e^{ia \cdot p_{(m)}} - 1\right) \tag{S2}$$

where $\alpha = k \cdot \Delta h \cdot (n-1)$, and $k = 2\pi/\lambda$ is the wave number and n is the real part of the refractive index of SC1827. This assumes patterning the CACDL on a clear, non-absorbing substrate such as glass.

The diffracted light field at the image plane (X'-Y') with distance $Z=d$ is described by the Fresnel transformation [1-3]:

$$U(x',\lambda,d) = \frac{e^{ikd}}{i\lambda d} \cdot \int g_{illum}(x) \cdot T(x) \cdot e^{i\frac{k}{2d}(x'-x)^2} dx \tag{S3}$$

in which $K_{(m)} = e^{ia \cdot p_{(m)}} - 1$. An on-axis unit-amplitude plane-wave illumination light was assumed $g_{(m)} = 1$.

The refractive index of the CACDL material SC1827 positive photoresist is measured by Woollam Ellipsometer. The measurement result (real part) from 400nm to 800nm is plotted in Fig. S2.

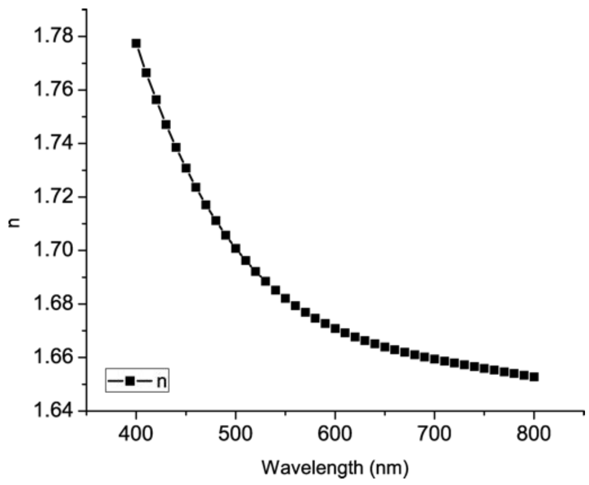

Figure S2 | Refractive index of SC1827 material measured by ellipsometer.

2. Optimization algorithm

Direct-binary-search (DBS) is an iterative algorithm. It is explored to search for the optimal solution of the CACDL height distribution [1,2]. To begin with, we need to define the figure-of-merit (FOM) to be maximized (see the next section). At first, an initial guess of height distribution is generated (usually a random distribution). In one iteration, all grooves are perturbed in a pre-designed manner (a random sequence). A positive unit perturbation ($+\Delta h$) is tried. If the updated FOM is increased, then this perturbation is kept, otherwise a negative unit perturbation ($-\Delta h$) is applied to this groove. If the new FOM is calculated to increase, then this negative perturbation is kept, otherwise it proceeds to the next groove. The guessed height distribution is updated accordingly. One iteration stops when all grooves are traversed. Termination conditions guarantee convergence, such as a maximum number of total iterations or a minimum FOM improvement threshold between two iterations. An algorithm flow chart is shown in Fig. S3.

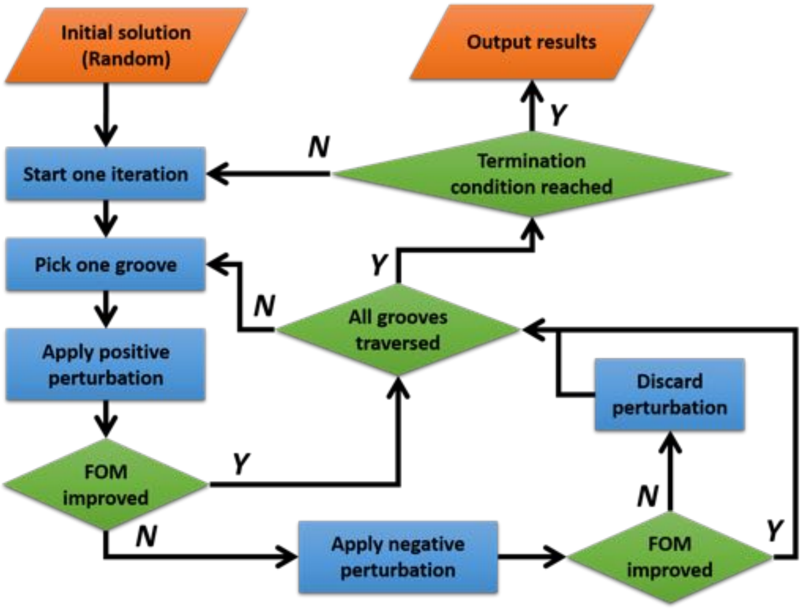

Figure S3 | Flow chart of the DBS algorithm to optimize the CACDL designs.

3. Figure-of-merit (FOM)

The FOM considered in the DBS-based optimization is defined by:

$$FOM = \frac{\sum_{i=1}^{N} \omega_i \cdot \mu_i}{N} - 10 \cdot \frac{\sum_{i=1}^{N} \omega_i \cdot \varepsilon_i}{N} \quad \text{(S4)}$$

The first term in Eq. S4 represents weighted efficiency averaged over totally N wavelength samples. The second term is the weighted normalized absolute difference (between the simulated and the target functions) over N wavelength samples. A factor 10 is introduced to balance these two term. The weighting coefficients ω_i balance contributions from different wavelength samples. Proper selection of this group of parameters is critical to achieve the optimal solution, which will be shown in the following section.

The efficiency μ_i and the normalized absolute difference ε_i in Eq. S4 are expressed by:

$$\mu_i = \frac{\int_{x'_{\min}}^{x'_{\max}} I_i(x') \cdot T_i(x') dx'}{\int_{x'_{\min}}^{x'_{\max}} I_i(x') dx'} \quad \text{(S5)}$$

$$\varepsilon_i = \frac{\int_{x'_{\min}}^{x'_{\max}} \left| normalize(I_i(x')) - T_i(x') \right| dx'}{\int_{x'_{\min}}^{x'_{\max}} dx'} \quad \text{(S6)}$$

Here, $I_i(x')=|U(x',\lambda)|^2$ is simulated light intensity distribution (along X' direction in Fig. S1) at the image plane for the ith wavelength sample. $T_i(x')$ is the target function for the ith wavelength sample. $x'_{\min}$ and $x'_{\max}$ delimit the integration range from the leftmost to the rightmost of the CACDL design.

As the first-order approximation of a focusing point-spread-function (PSF), the target function is defined as a Gaussian function centered at $(x'_{\min} + x'_{\max}) / 2$ with full-width-at-half-maximum (FWHM) W_i determined by the far-field diffraction limit.

$$T_i(x') = \exp\left\{ - \frac{\left[x' - \left(\frac{x'_{\min} + x'_{\max}}{2} \right) \right]^2}{(\frac{W_i}{2})^2} \right\} \quad \text{(S7)}$$

$$W_i = \frac{\lambda_i}{2 \cdot NA} \quad \text{(S8)}$$

$$NA = \sin\left[\tan^{-1}\left(\frac{L_X/2}{f} \right) \right] \quad \text{(S9)}$$

In Eqs. S7-S9, λ_i is the ith wavelength sample. L_X is the total length of the CACDL design in X direction (see Fig. S1) and f is the designed focal length (gap between the CACDL and the image plane). Three $T_i(x')$ examples for λ_1=460nm, λ_2=540nm and λ_3=620nm are plotted in Fig. 2(a).

Note that the efficiency μ_i defined in Eq. S5 is for the purpose of optimization only. When evaluating the devices' performances, an optical efficiency η_i, defined as ratio of the energy integrated within the zero-order lobe to the total energy, is utilized:

$$\eta_i = \frac{\int_{-\frac{w}{2}}^{+\frac{w}{2}} I_i(x')dx'}{\int_{x'_{\min}}^{x'_{\max}} I_i(x')dx'} \tag{S10}$$

An example schematically depicting the integration region with width w (gray-shaded) using a Bessel function of the first kind is plotted in Fig. S4.

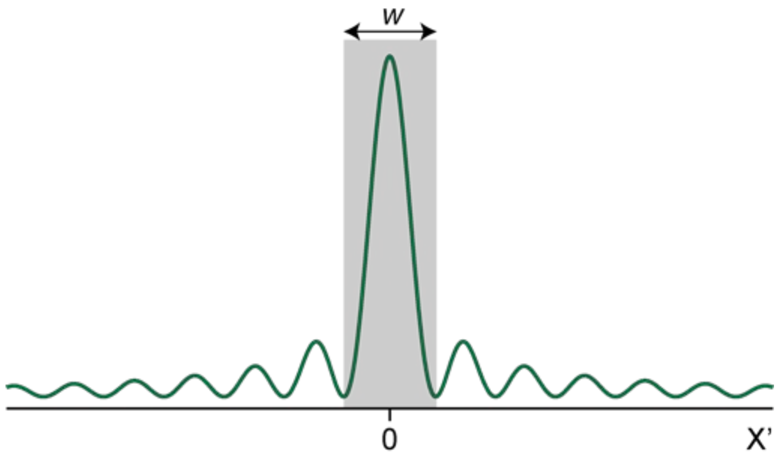

Figure S4 | Schematic illustration of the integration region used in optical efficiency η_i definition.

4. Design and optimization parameters

4.1 CACDLs for three discrete wavelengths (apochromat)

The optimal combinations of weighting coefficients ω_i in Table S1 are selected among a number of tried candidates.

Table S1 | Parameters for the CACDL designs (#1 – #3)

	Design	CACDL#1	CACDL#2	CACDL#3
**Design parameters**	*Number of grooves*	2800		280
	Groove width	3μm		
	Total length	8.4mm		0.84mm
	Maximum height	3000nm		
	Aspect ratio	1 : 1		
	Number of levels	61		
	Unit height	50nm		
	Focal length	120mm	25mm	10mm
	NA	0.035	0.166	0.042
	Material	SC1827		
	Wavelengths	460nm; 540nm; 620nm		
**Optimization parameters**	*Weighting coefficients* $\omega_1 : \omega_2 : \omega_3$	0.75 : 0.97 : 1.00	0.90 : 1.00 : 0.98	0.95 : 0.98 : 1.00
	Maximum number of iterations	1000		100
	Minimum FOM improvement threshold	1e-5		

4.2 CACDL over visible spectrum (super-achromat)

Since more wavelength samples are involved in the broadband CACDL designs, it adds more complexities and difficulties to optimization. The finally optimized design is achieved via two steps with different weighting coefficients ω_i, and the optimization result from the first step is imported as the initial solution in the second step. The evolutions of FOM versus times of iterations in these two steps are plotted in Fig. S5.

Table S2 | Parameters for the CACDL design (#4)

	Design	CACDL#4
**Design parameters**	*Number of grooves*	2500
	Groove width	3μm
	Total length	7.5mm
	Maximum height	3000nm
	Aspect ratio	1 : 1
	Number of levels	61
	Unit height	50nm

	Focal length	280mm
	NA	0.013
	Material	SC1827
	Wavelengths	450nm; 455nm; 460nm; 465nm; 470nm; 475nm; 480nm; 485nm; 490nm; 495nm; 500nm; 505nm; 510nm; 515nm; 520nm; 525nm; 530nm; 535nm; 540nm; 545nm; 550nm; 555nm; 560nm; 565nm; 570nm; 575nm; 580nm; 585nm; 590nm; 595nm; 600nm; 605nm; 610nm; 615nm; 620nm; 625nm; 630nm; 635nm; 640nm; 645nm; 650nm; 655nm; 660nm; 665nm; 670nm; 675nm; 680nm; 685nm; 690nm
**Optimization parameters**	*Weighting coefficients in step 1* $\omega_1 : \omega_2 : \omega_3$	1.00 : 1.00
	Weighting coefficients in step 2 $\omega_1 : \omega_2 : \omega_3$	0.88 : 0.88 : 0.88 : 0.95 :0.95 : 0.95 : 0.96 : 0.96 : 0.96 : 0.97 : 0.97 : 0.97 : 0.98 : 0.98 : 0.98 : 0.97 : 0.97 : 0.97 : 0.98 : 0.98 : 0.98 : 0.98 : 0.98 : 0.98 : 0.94 : 0.94 : 0.94 : 0.93 : 0.93 : 0.93 : 0.93 : 0.93 : 0.93 : 0.94 : 0.94 : 0.94 : 0.95 : 0.95 : 0.95 : 0.95 : 0.95 : 0.95 : 0.96 : 0.96 : 0.96 : 0.98 : 0.98 : 0.98 : 1.00
	Maximum number of iterations	1000
	Minimum FOM improvement threshold	1e-5

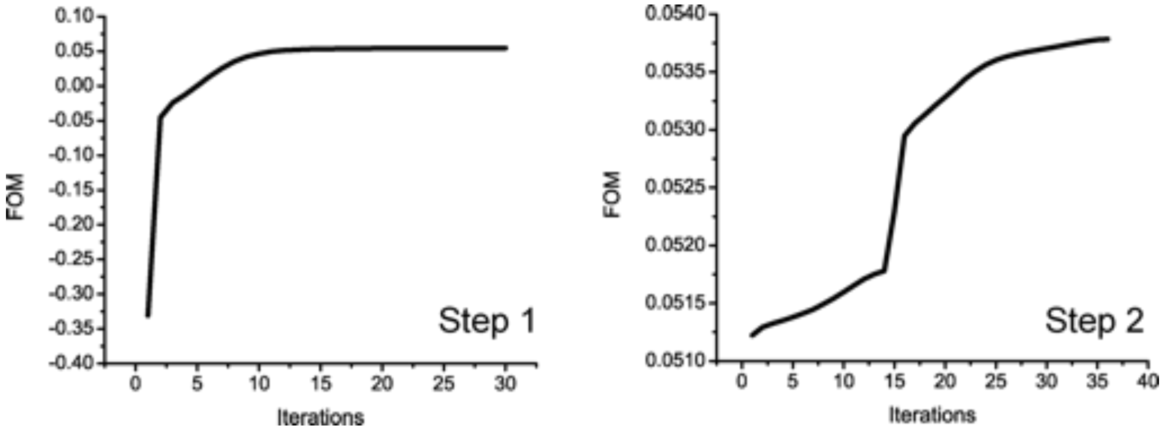

Figure S5 | FOM versus times of iterations for the two steps towards the optimal CACDL#4 design.

5. Optimized CACDL designs

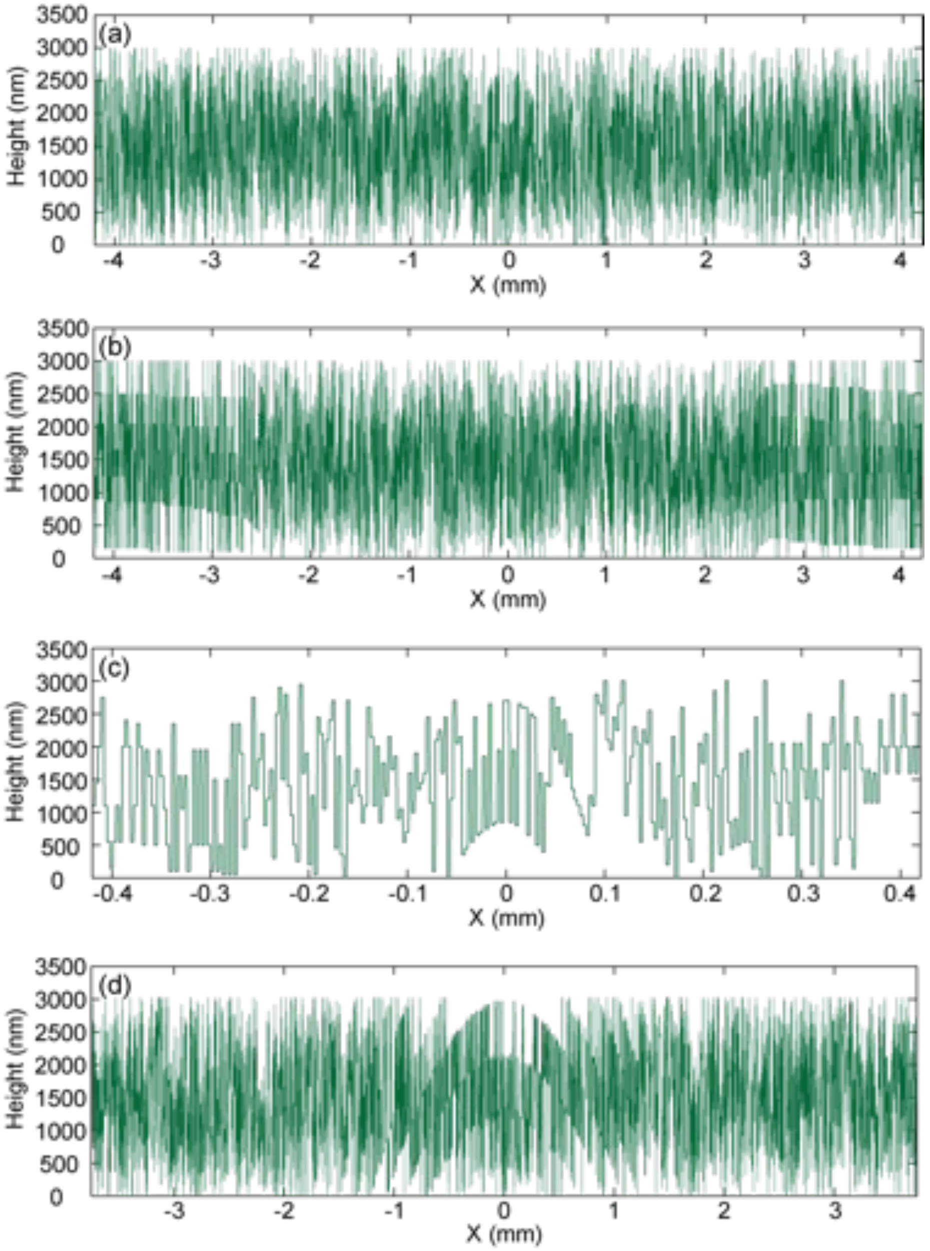

Figure S6 | Optimized height profiles of the CACDL designs: (a) CACDL#1 with 2800 3μm-wide grooves for focusing at 120mm. (b) CACDL#2 with 2800 3μm-wide grooves for focusing at 25mm. (c) CACDL#3 with 280 3μm-wide grooves for focusing at 10mm. (d) CACDL#4 with 2500 3μm-wide grooves for focusing at 280mm. (a), (b) and (c) are designed for three discrete wavelengths 460nm, 540nm and 620nm; (d) is designed for broadband spectrum from 450nm to 690nm.

6. Device fabrication

In gray-scale lithography, the write head scans through the sample surface and the exposure dose at each point is modulated with different gray-scales [2,4]. Most photoresist is characterized by a contrast curve. Different depths in accord with different exposure doses are achieved after development. Greater dose leads to deeper feature. Before patterning structures, it is needed to calibrate this contrast curve. Figure S7(a) gives a typical calibration plot, based upon which the exposure gray-scale at each point is given. The surface roughness measurement (max-min height ~13.5nm) by AFM demonstrates the excellent smoothness of surface after gray-scale fabrication.

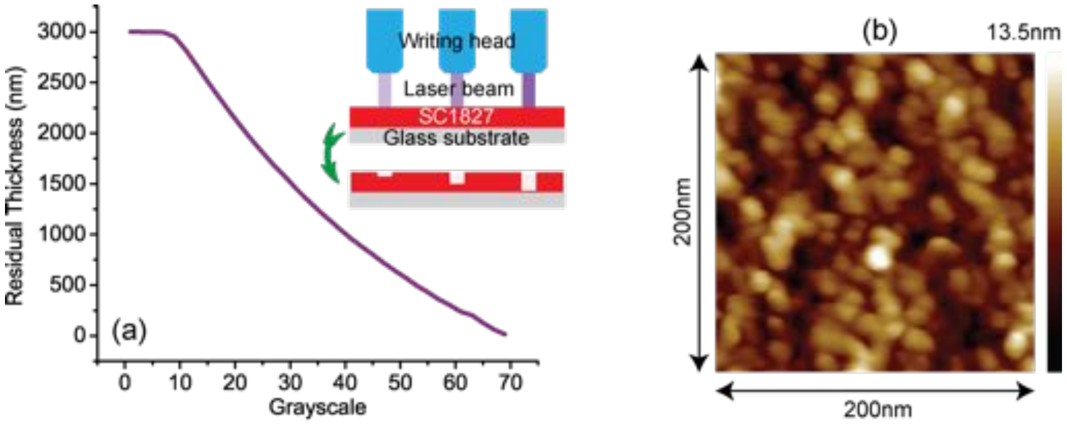

Figure S7 | (a) Calibration curve of the SC1827 photoresist at laser power of 26mW and duration factor of 50%. Inset: schematic illustration of gray-scale lithography on a positive photoresist. (b) Surface roughness measurement of the fabricated CACDL by AFM.

Fabrication procedure:

1) RCA clean a 3-inch soda-lime glass substrate.
2) Spin coat SC1827 photoresist (positive), 60 seconds @ 1800 rpm. This leads to a uniform film of ~3.4μm thick.
3) Soft bake in an oven, 30 minutes @ 110°C.
4) Dehydration in fume hood for ~24 hours.
5) Gray-scale exposure by the Heidelberg microPG101 [5] with 3μm-mode. Power: 26mW; duration factor: 50 %; mode: uni-directional; scan-scale: 2.74MHz.
6) Development in AZ 1:1 solution for 2 minutes.
7) Rinse in DI water for 1 minute. Dry the sample by blowing Nitrogen.

7. Setups

7.1 PSF measurement setup

A single-mode-fiber (SMF) tip, connected to an Ocean Optics Jaz spectrometer [6] and mounted onto a motor-actuated two-axis stage, is utilized to measure the PSFs of CACDL designs at different wavelengths. The SMF has core diameter of ~8μm and is placed precisely at the focal plane (gap is *f*). The sample is aligned to be perpendicular to the optical path while the fiber is in line with the optical path. The spectrometer and two actuators (Thorlabs Z825B) are controlled via a custom-built LabView routine.

An achromatic biconcave lens expands the white-laser beam from the SuperK EXTREME EXW-6 source [7] and the SuperK VARIA filter [8]. Two concave mirrors of different focal lengths further expand and collimate the beam. The source is set to have a power level of 50%.

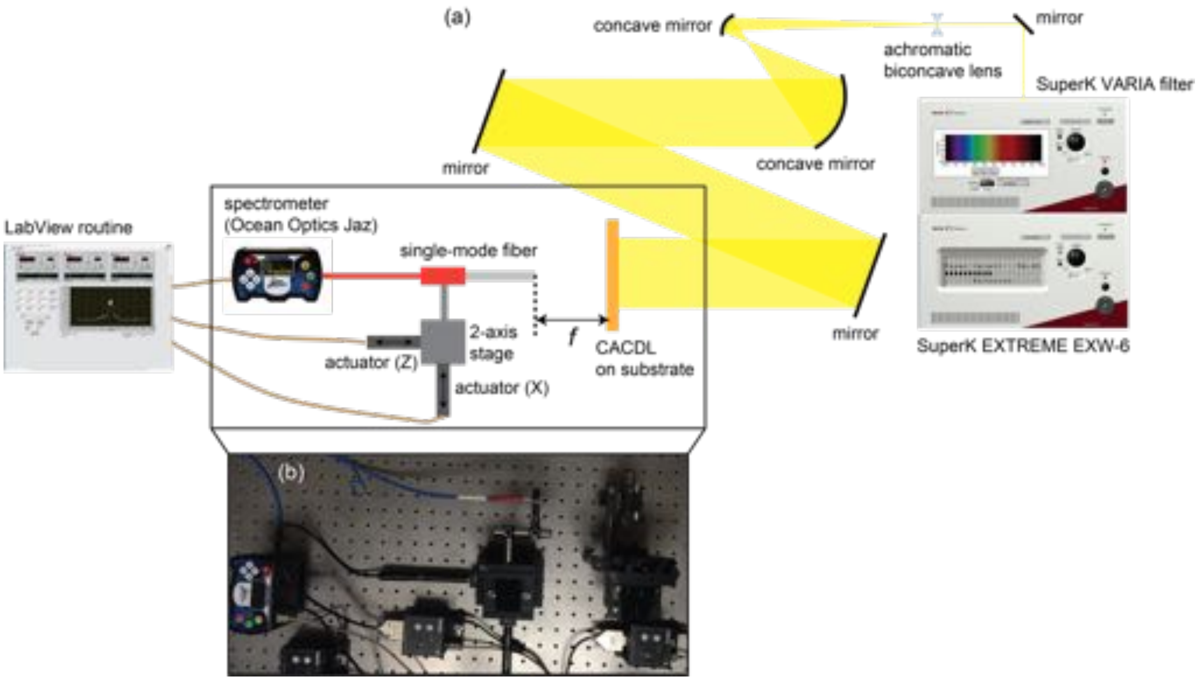

Figure S8 | (a) Schematic of the setup measuring PSFs of the CACDL designs. (b) Photograph of part of the setup (black box in (a))

The point-spread-function (PSF) at wavelength λ can be derived from the following equation:

$$PSF(x',\lambda) = \frac{I(x',\lambda) - I_{dark}(\lambda)}{I_{ref}(\lambda) - I_{dark}(\lambda)} \tag{S11}$$

In Eq. S11, $I(x',\lambda)$ is the intensity measured by spectrometer (Fig. S8(a)) at position x' and wavelength λ. And $I_{ref}(\lambda)$ is the reference spectrum when the beam passes through a bare glass substrate coated with uniform unpatterned photoresist SC1827. The photoresist is prepared at the same condition with the same film thickness. $I_{dark}(\lambda)$ is the dark measurement without light illumination.

7.2 Other setups

In the setup for studying light propagation around the focal point, a commercial monochrome board CMOS camera

(The Imaging Source DMM22BUC03-ML) replaces the fiber-spectrometer assembly in Fig. S8(a). The rest of the setup remains the same. This camera has pixel size 6μm×6μm. It is mounted onto a one-axis motorized motion stage (Z direction).

In the polarization test experiment, a linear polarizer (Edmund Optics) is inserted into the optical path. Orthogonal polarization states are realized by rotating the polarizer by 90°.

8. Spectra

Figure S9 summarizes the spectra used in this work. They are measured by the same single-mode fiber and the Ocean Optics Jaz spectrometer (Fig. S8(a)). The light source is a high-power super-continuum laser [7]. A reconfigurable filter SuperK VARIA [8] is used to select spectrum of interest in the visible band. The minimum achievable bandwidth is 10nm [8].

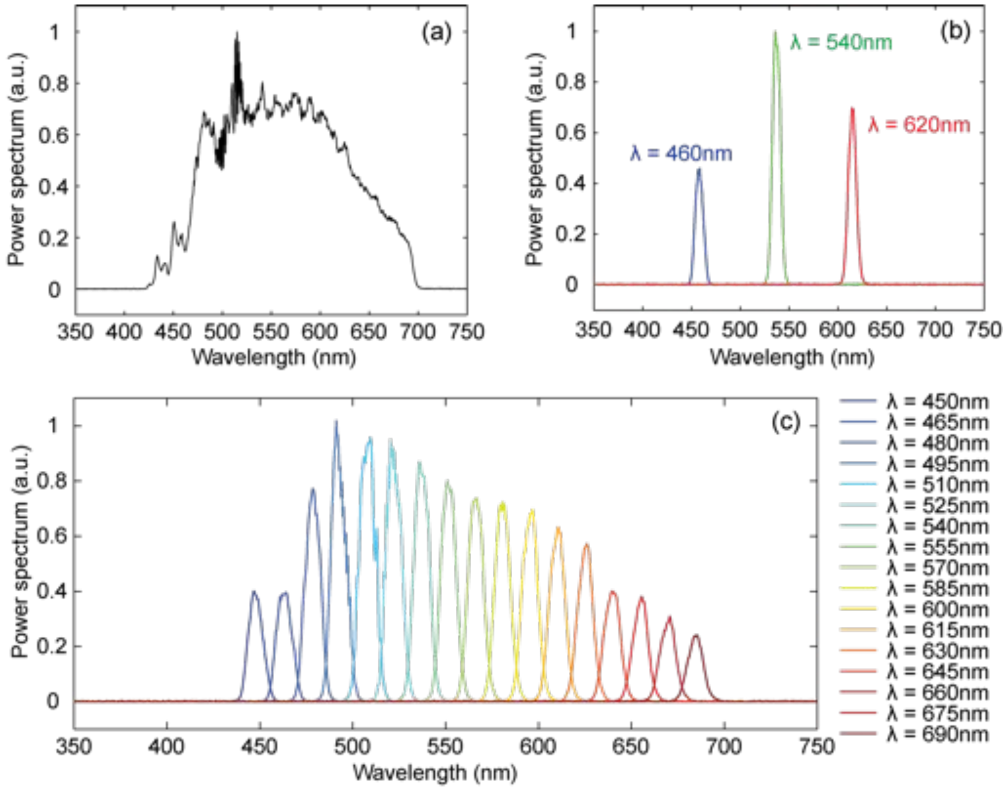

Figure S9 | (a) Broadband spectrum for PSF measurements. VARIA filter is set to be from 400nm to 700nm. (b) Three narrow spectra of interest for imaging the CACDLs#1–#3 on a monochrome digital camera. Central wavelengths are 460nm, 540nm and 620nm, with bandwidth of 10nm. (c) Seventeen narrow spectra of interest for imaging the CACDL#4 on a monochrome digital camera. Central wavelengths are from 450nm to 690nm with 15nm spacing and bandwidth of 10nm. All the spectra are normalized.

9. Analysis

9.1 Effect of the number of CACDL periods

The multi-wavelength PSFs of the CACDL#1 design is measured when the fabricated device is illuminated by different beam dimensions. The beam size is adjusted by an iris. Since the CACDL#1 design is fabricated with 5 CACDL periods padded in X direction, the effect of the number of CACDL periods is tested with 0.5, 1, 2, 3, 4 and 5 periods. As shown in Fig. S10(a), the optical efficiencies start to drop when the number of periods is less than 2. However, the optical efficiencies still remain reasonably high when the beam size only covers one CACDL period. In addition, the PSFs at three designed wavelengths don't experience any obvious lateral shifts for the 0.5-5 range of the number of the CACDL periods (see Figs. S10(b)-(d)). This indicates that periodic padding is good but not necessary for our CACDL designs to work properly.

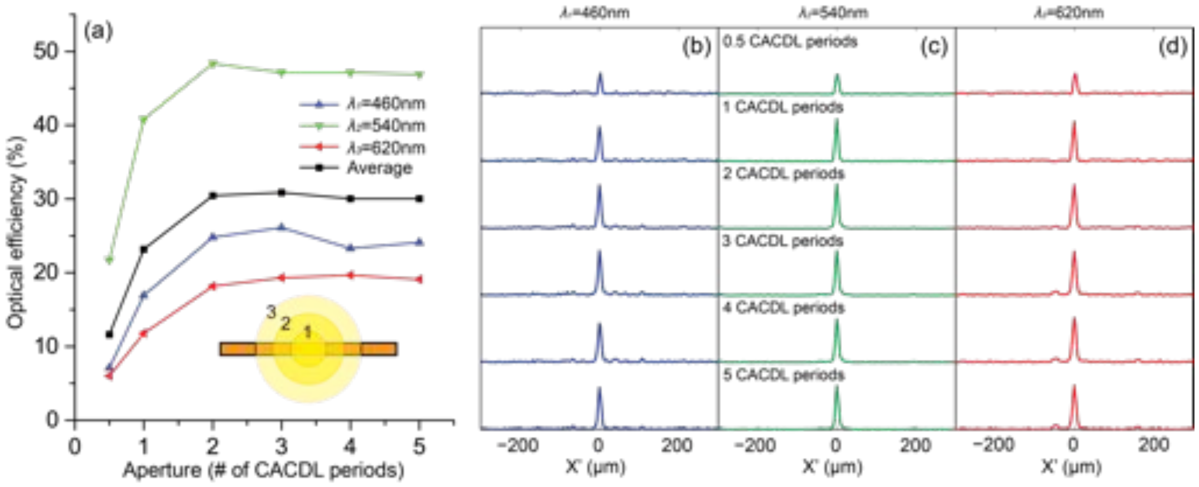

Figure S10 | (a) Measured optical efficiencies when the fabricated CACDL#1 design is illuminated by beam covering different number of CACDL periods. Inset: schematic illustrating different beam sizes covering different number of CACDL periods. Measured PSFs at *f*=120mm when the CACDL is illuminated by different beam dimensions for wavelengths of (b) 460nm, (c) 540nm and (d) 620nm. Only the X'=-300μm to X'=300μm segment is displayed here.

9.2 Effect of the refractive index measurement error

A Woollam Spectroscopic Ellipsometer is used to measure the refractive index of the CACDL material at different wavelengths (see Fig. S2). A thin film of SC1827 spun coated on Si wafer works as the sample. However, this measurement may experience some errors which eventually lead to compromised CACDL performances since according to Section 1 in this Supplementary Material the phase shift imparted by the microstructure at wavelength λ is a function of refractive index $n(\lambda)$. Figure S11 numerically studies this effect of the refractive index measurement error. The plots demonstrate that the devices are relatively tolerable to this measurement error since within the ±0.1 error (equivalent to 1.6~1.8 range for a true value n=1.7) the average optical efficiencies maintain acceptably high and the averaged absolute lateral focal spot shifts are still strongly suppressed.

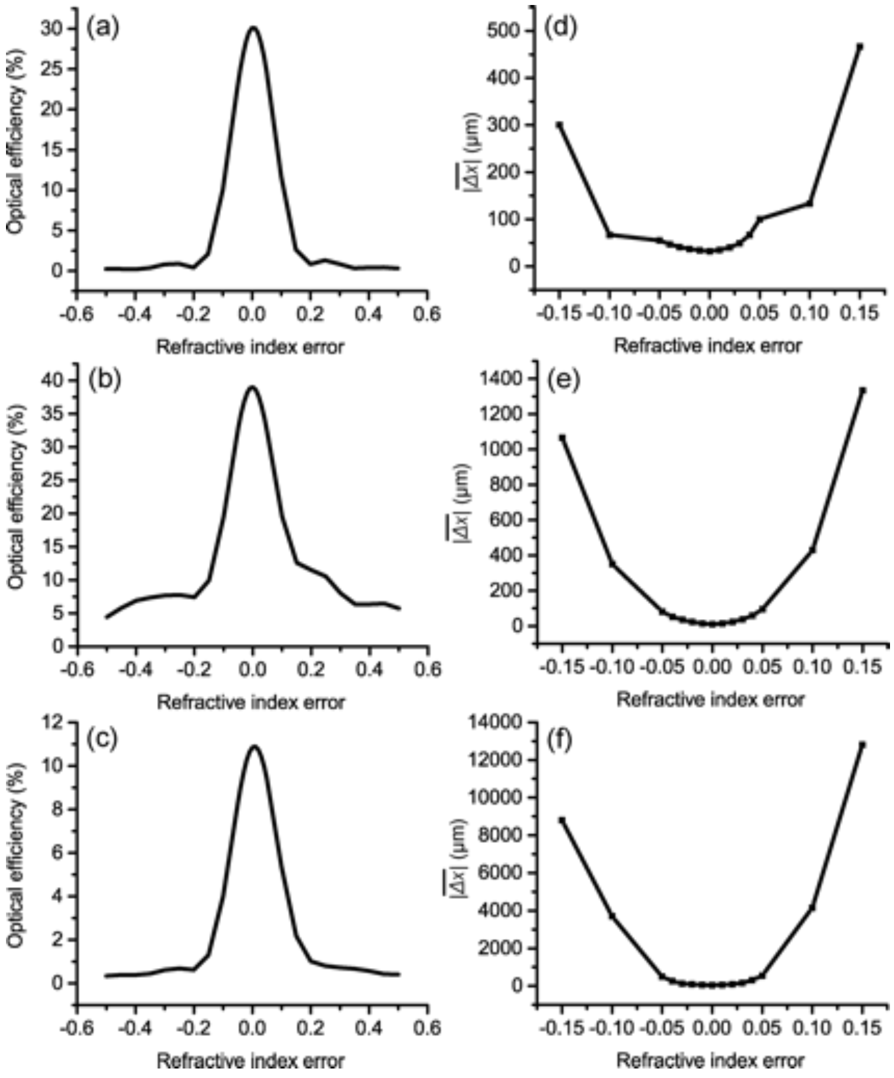

Figure S11 | Numerical studies on the effect of the refractive index measurement errors: (a)-(c) plots of the average optical efficiencies versus different refractive index errors (ranging from -0.5 to +0.5); (d)-(f) plots of the averaged absolute lateral focal spot shifts versus different refractive index errors (ranging from -0.5 to +0.5). (a) and (d) CACDL#1 design; (b) and (e) CACDL#3 design; (c) and (f) CACDL#4 design.

10. Diffractive lens optimized for single wavelength

Figure S12 gives the result of a diffractive lens optimized for just one single wavelength. Here the lens is pixelated (Δ=3μm) and has maximum height of 3μm. It is comprised of 2800 pixels and is designed for focusing at f=120mm with numerical aperture of 0.035. DBS-based algorithm optimizes single wavelength focusing at λ=540nm. Its chromatic aberration (axial focal spot displacement Δf) over the visible spectrum is also plotted, showing shift from +27mm to -25mm.

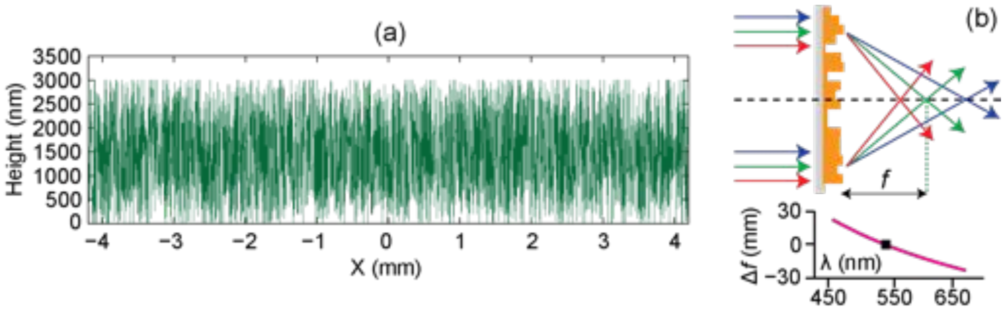

Figure S12 | A conventional diffractive lens optimized for single wavelength focusing (λ=540nm). (a) The optimized height profile with 3μm maximum height and 3μm pixel size. (b) Schematic showing its chromatic aberration and the calculated plot of the axial focus shift versus wavelength.

11. CACDL with higher optical efficiency

To explore the ultimate performance of the CACDL design, Figure S13 summarizes the simulation results of a CACDL with much higher optical efficiency by increasing the aspect ratio of the microstructure from 1:1 to 3.3:1 and increasing the number of grooves from 2800 to 4000. The device still has groove width of Δ=3μm, but the maximum height is increased to H=10μm and totally 201 quantization levels with unit height of 50nm. The augmented number of quantization levels (compared to the 61 levels for the CACDL designs #1-#4 in the main text) and the augmented number of grooves add to much more degrees of design freedom, therefore leads to improved optical efficiency. This was also previously observed in our initial work on designing multi-wavelength holograms [1].

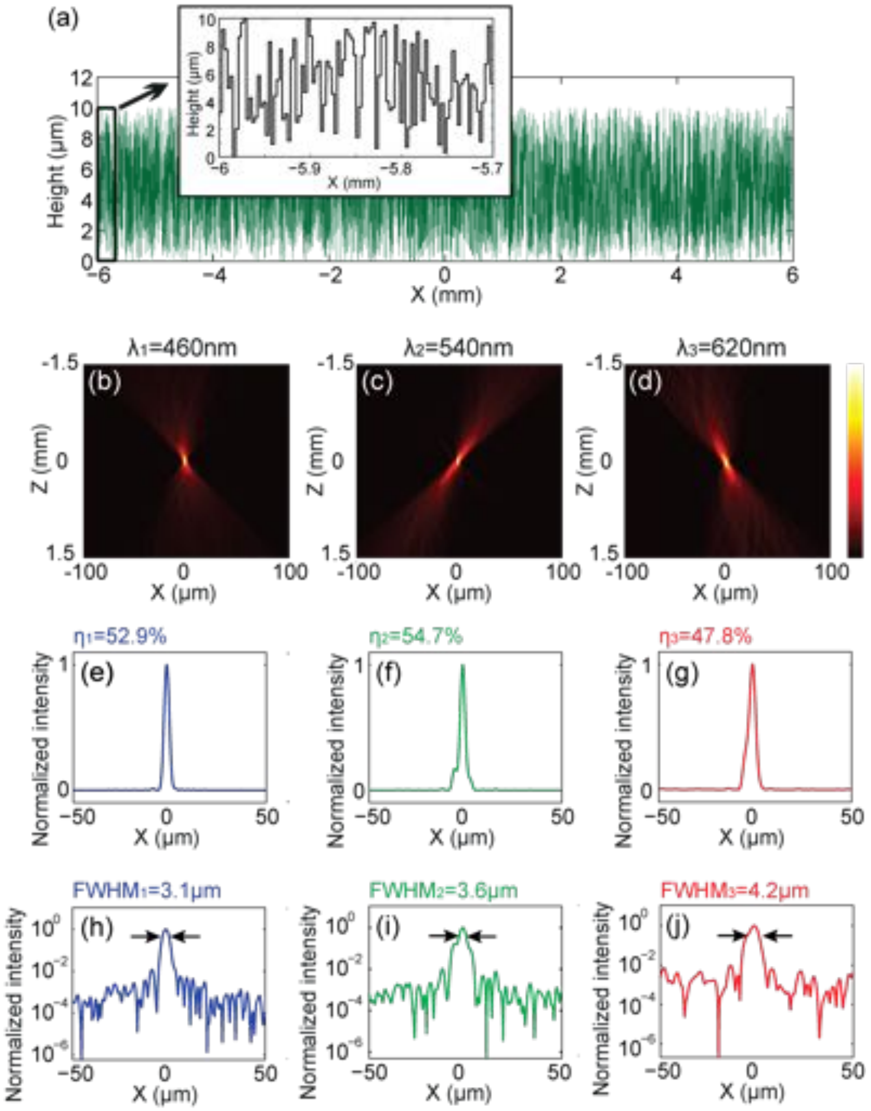

Figure S13 | An optimized CACDL design with higher optical efficiency. (a) Its optimized height profile. It has groove width Δ=3μm, maximum height H=10μm and the total number of grooves N=4000. Inset: the magnified view of the first 0.3mm-long section. Simulated intensity distributions along light propagation within the region from X=-100μm to X=+100μm for three discrete wavelengths λ=460nm (b), 540nm (c) and 620nm (d). Linear-scale plots of the focal spots at the focal plane within the region from X=-50μm to X=+50μm for three wavelengths λ=460nm (e), 540nm (f) and 620nm (g). Logarithm-scale plots of the focal spots at the focal plane for three wavelengths λ=460nm (h), 540nm (i) and 620nm (j).

For simplicity, the CACDL was optimized for three discrete wavelengths (λ=460nm, 540nm and 620nm, apochromatic like CACDLs #1-#3 in the main text). The optimized height profile is plotted in Fig. S13(a). The designated focal length is 80mm, resulting in a numerical aperture of 0.075. It has 4000 grooves, which makes one period of CACDL 12mm long in the X direction. The light intensity distributions along propagation (Z direction) for three wavelengths are shown in Figs. S13(b) – (d). Note that Z=0 corresponds to the nominal focal plane and only the central part from X=-100μm to X=+100μm is shown for clarity. The normalized intensity profiles (or PSFs) at the focal plane are given in linear scale in Figs. S13(e) – (g) and in logarithm scale in Figs. S13(h) – (j). The optical efficiencies of collecting light over 12mm dimension to narrow focal spots are 52.9%, 54.7% and 47.8% for three wavelengths, respectively. The average efficiency is 51.8%. The FWHMs of the focal spots are 3.1μm, 3.6μm and 4.2μm, respectively.

12. CACDL wavefronts

The wavefronts of the CACDL designs simulated by scalar diffraction formulation are plotted in Figs. S14 and S15. The wavefront RMSs in the region of focus are calculated (in terms of phase pi) to quantify spherical aberration present in the focusing device.

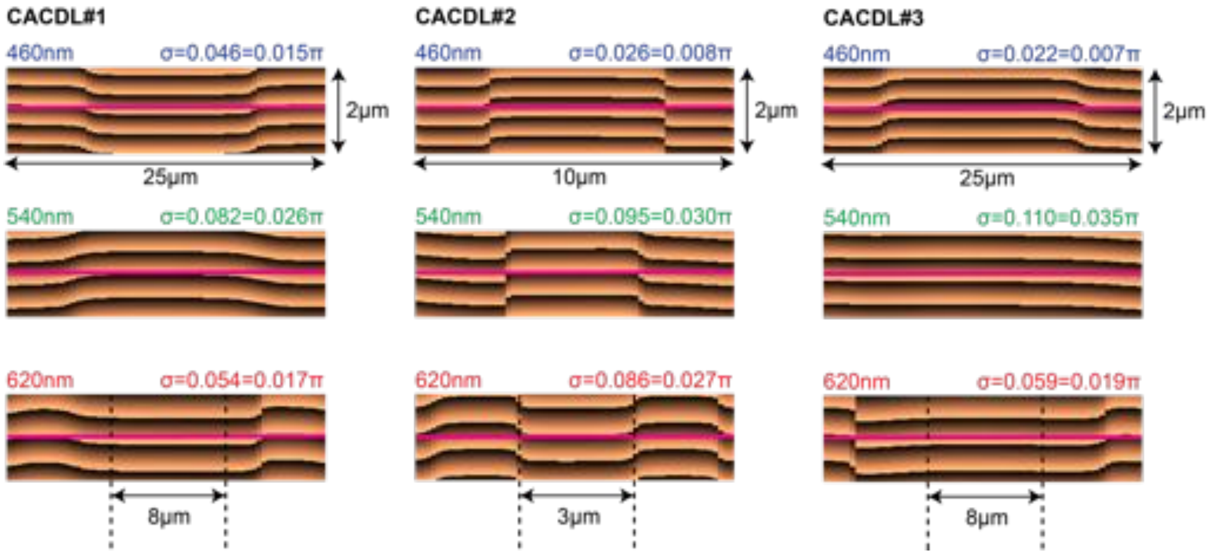

Figure S14 | Simulated wavefronts of CACDLs#1-3 in the vicinity of focus. The region of focus is defined by black dashed lines. The wavefront RMSs are calculated accordingly.

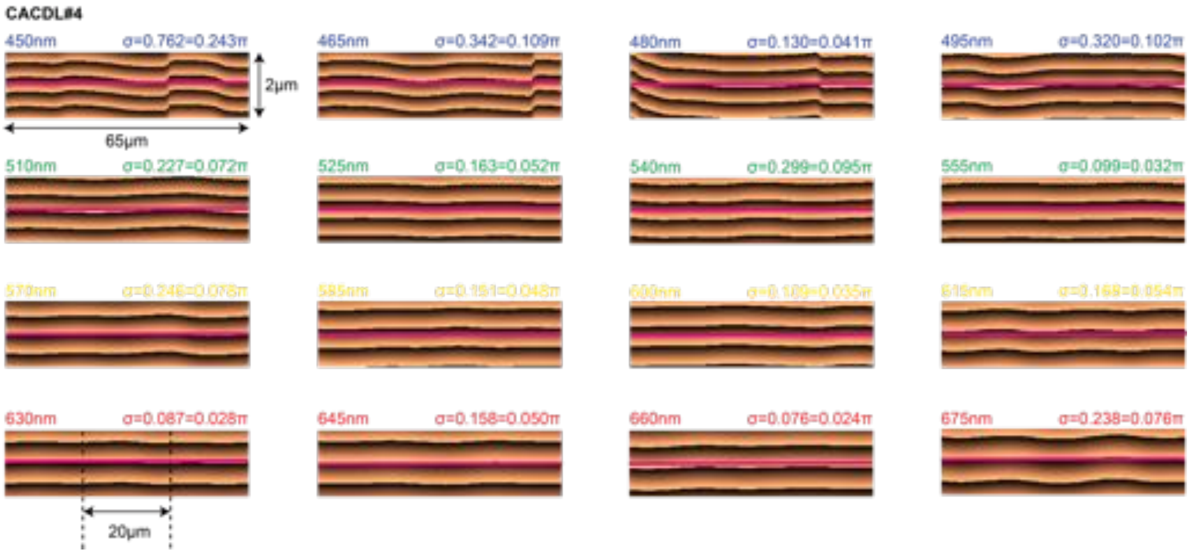

Figure S15 | Simulated wavefronts of CACDL#4 in the vicinity of focus. The region of focus is defined by black dashed lines. The wavefront RMSs are calculated accordingly.

References:

[1] G. Kim, J. A. Dominguez-Caballero, and R. Menon, "Design and analysis of multi-wavelength diffractive optics," *Opt. Express* **20**, 2814-2823 (2012).

[2] P. Wang, J. A. Dominguez-Caballero, D. J. Friedman, and R. Menon, "A new class of multi-bandgap high-efficiency photovoltaics enabled by broadband diffractive optics," *Prog. Photovolt: Res. Appl.* (2014) DOI: 10.1002/pip.2516.

[3] Goodman, J. W. *Introduction to Fourier Optics.* (Roberts and Company Publishers, 2005).

[4] K. Reimer, H. J. Quenzer, M. Jurss, and B. Wagner, "Micro-optic fabrication using one-level gray-tone lithography," *Proc. SPIE* **3008**, 279-288 (1997).

[5] Data sheet of Heidelberg μPG 101: http://www.himt.de/index.php/upg-101.html

[6] Ocean Optics Jaz spectrometer: http://oceanoptics.com/product-category/jaz-series/

[7] SuperK EXTREME EXW-6 source from NKT Photonics: http://www.nktphotonics.com/superkextreme

[8] SuperK VARIA filter from NKT Photonics: http://www.nktphotonics.com/superk_varia
